# Deep learning algorithm using bispectrum analysis energy feature maps based on ultrasound radiofrequency signals to detect breast cancer

**DOI:** 10.3389/fonc.2023.1272427

**Published:** 2023-12-07

**Authors:** Qingmin Wang, Xiaohong Jia, Ting Luo, Jinhua Yu, Shujun Xia

**Affiliations:** ^1^School of Information Science and Engineering, Fudan University, Shanghai, China; ^2^Ruijin Hospital, School of Medicine, Shanghai Jiao Tong University, Shanghai, China

**Keywords:** ultrasound radiofrequency signals, bispectrum analysis, breast cancer, deep learning, weight sharing, attention mechanism, similarity constraint, multiple feature maps

## Abstract

**Background:**

Ultrasonography is an important imaging method for clinical breast cancer screening. As the original echo signals of ultrasonography, ultrasound radiofrequency (RF) signals provide abundant tissue macroscopic and microscopic information and have important development and utilization value in breast cancer detection.

**Methods:**

In this study, we proposed a deep learning method based on bispectrum analysis feature maps to process RF signals and realize breast cancer detection. The bispectrum analysis energy feature maps with frequency subdivision were first proposed and applied to breast cancer detection in this study. Our deep learning network was based on a weight sharing network framework for the input of multiple feature maps. A feature map attention module was designed for multiple feature maps input of the network to adaptively learn both feature maps and features that were conducive to classification. We also designed a similarity constraint factor, learning the similarity and difference between feature maps by cosine distance.

**Results:**

The experiment results showed that the areas under the receiver operating characteristic curves of our proposed method in the validation set and two independent test sets for benign and malignant breast tumor classification were 0.913, 0.900, and 0.885, respectively. The performance of the model combining four ultrasound bispectrum analysis energy feature maps in breast cancer detection was superior to that of the model using an ultrasound grayscale image and the model using a single bispectrum analysis energy feature map in this study.

**Conclusion:**

The combination of deep learning technology and our proposed ultrasound bispectrum analysis energy feature maps effectively realized breast cancer detection and was an efficient method of feature extraction and utilization of ultrasound RF signals.

## Introduction

Breast cancer is the most common malignancy among women, ranking first in the global incidence rate and mortality rate of female cancer (1). In 2020, there will be around 2.3 million new cases and 685,000 deaths of breast cancer worldwide (2). The incidence of breast cancer has already surpassed lung cancer and become the largest cancer in the world (3). Breast cancer screening is helpful in the early detection of breast cancer. Through early intervention, it helps reduce the mortality of breast cancer and improve the life quality of those patients.

Among many imaging diagnostic methods of breast cancer, such as ultrasonography, magnetic resonance imaging, mammography, and electronic computed tomography (4), ultrasonography is an important imaging modality for screening breast cancer in clinic. It is not affected by the density of breast tissue (5, 6), easy to operate, relatively inexpensive, radiation-free, and widely used in the examination of breast cancer. At present, imaging is widely used in clinical practice and ultrasound diagnostic of breast cancer mainly relies on traditional ultrasound images (7, 8), such as grayscale images. Although grayscale images can clearly display the anatomical structure of the breast and target lesions, they are obtained after filtering, dynamic range adjustment, and a series of other post-processing from the original ultrasound radio frequency (RF) echo signals. In the process of enhancing the required visual information, it loses some high-frequency components and other information that may be valuable for cancer diagnosis. Research has confirmed that different ultrasound grayscale reconstruction algorithms based on RF signals have a significant impact on the classification performance of benign and malignant breast tumors (9). Therefore, using raw RF signals to construct computer-aided diagnostic system is more conducive to helping radiologists improve diagnostic efficiency.

Some studies have utilized deep learning techniques to process medical ultrasound RF signals. Liu et al. (10) used a convolutional neural network (CNN) to analyze RF signals and distinguish between benign and malignant thyroid nodules, with an accuracy of 96.2%. Luo et al. (11) designed a multichannel CNN to process RF signals and then screen for osteoporosis. Compared with traditional sound speed screening, the accuracy was significantly improved, reaching 83.05%. Xiao et al. (12) used the proposed deep learning method to track the displacement of blood vessel walls from RF signals, improving the accuracy of vessel wall displacement tracking. Yoon et al. (13) utilized a deep learning method to efficiently reconstruct B-Mode ultrasound image from RF signals. Qiao et al. (14) applied the YOLOv3 network to process RF signals and detect breast calcification.

Many CNN-based computer-aided diagnostic systems have been employed in the differentiation of benign and malignant breast tumors (9, 15–17), but there are relatively few studies on deep learning methods for processing RF signals to achieve breast cancer detection. Kim et al. (18) used CNN to process multiple parametric images generated from RF signals for breast benign and malignant classification. In their research, the highest classification accuracy of the network models based on entropy images, phase images, attenuation images, and ultrasound grayscale images were 82.00%, 74.50%, 74.50%, and 79.00%, respectively. The highest accuracy and recall of combining multiple parametric images were 83.00% and 92.24%, respectively. Compared with the traditional method of only using ultrasound grayscale image in the network model, the use of multiple parameter images improved classification accuracy and recall by 5.5% and 11.6%, respectively. Extracting multiple parameters from RF signals and establishing a multifeature map system based on RF signals can help deep learning networks obtain more abundant sample information from the original echo signals.

In this study, we designed multiple feature maps of RF signals based on bispectrum analysis and combined them with an end-to-end neural network framework to extract valuable features for effective breast cancer detection. We first proposed bispectrum analysis energy feature maps, which were composed of different frequency components of RF signals. They were based on high-order spectral analysis, which has better time-frequency localization ability than the traditional power spectrum analysis method. Alqudah et al. (19) showed that the performance of the features extracted by the high-order spectrum analysis method was better than that of the low-order feature extraction methods, such as short-time Fourier transform and wavelet transform. The existence of higher-order cumulants enables higher-order spectral analysis to adapt to non-stationary local characteristics, such as spikes and abrupt changes in signals, when processing non-stationary signals. Ultrasound RF signals are a typical non-stationary signal, which has the characteristics of rapid instantaneous phase and frequency change and concentrated energy distribution and is very suitable for feature extraction using high-order spectral analysis.

According to the input of multiple feature maps, a weight sharing deep learning network framework was constructed. An appropriate backbone network was selected from five typical networks. Additionally, a feature map attention module and a similarity constraint module were designed to guide the network in learning feature maps and features that were advantageous for classification, accelerate network convergence, and improve the classification efficiency of breast tumors.

## Methods

### Patients

We collected 203 cases of breast cancer patients from Ruijin Hospital, Shanghai Jiao Tong University School of Medicine. Initially, 123 patients were collected, of which 73 had benign breast tumors and 50 had malignant breast tumors. These were divided into a training set (70%), a validation set (15%), and a test set 1 (15%). An additional independent test set 2 consisted of 80 patients, with a 50% distribution of benign and malignant breast tumors.

The patient data included continuous multiple frames of ultrasound RF signals and ultrasound grayscale images. All data were obtained using the Resona 7 ultrasound equipment of Mindray™. Two experienced radiologists examined the ultrasound grayscale images with marked tumor locations and selected a key frame of RF data that displayed clear imaging and encompassed the entire tumor area for each patient.

Pathological results served as the gold standard for distinguishing benign and malignant breast tumors in this study. The Ethics Committee of Ruijin Hospital, Shanghai Jiao Tong University School of Medicine, approved this research.

### Bispectrum analysis energy feature maps of ultrasound RF signals

The high-order spectral analysis method analyzes the spectral characteristics of a signal by introducing high-order statistics, such as third-order moments and fourth-order moments, reflecting the nonlinear characteristics and phase correlation of the signal. Assuming *x*_bs_(*n*_bs_) represents a certain RF signals sequence, where *n*_bs_=1, …,256 is the number of samples. Bispectrum of RF signals *x*_bs_(*n*_bs_) is defined as third-order cumulant *C*_3_ Fourier transform BS, where the third-order cumulant *C*_3_ is (20):


(1) $$C_{3}\left(n_{bs},\;k_{bs},\;l_{bs}\right)=\left[x_{bs}^{\;*}\left(n_{bs}\right)x_{bs}\left(n_{bs}+k_{bs}\right)x_{bs}\left(n_{bs}+l_{bs}\right)\right]\;$$

Among them, *k_bs_* and *l_bs_* are time delays, so the bispectrum BS of the RF signals is:


(2) $$\text{BS}\left({\text{f}}_{1},{\text{f}}_{2}\right)=\sum_{{\text{k}}_{bs}}\sum_{{\text{l}}_{bs}}{\text{C}}_{3}\left(n_{bs},\;k_{bs},\;l_{bs}\right){\text{e}}^{{-j 2\pi f}_{1}k_{bs}}{\text{e}}^{{-j 2\pi f}_{2}l_{bs}}$$

Among them, f_1_ and f_2_ represent the horizontal and vertical frequency axes.

**Figure 1:**
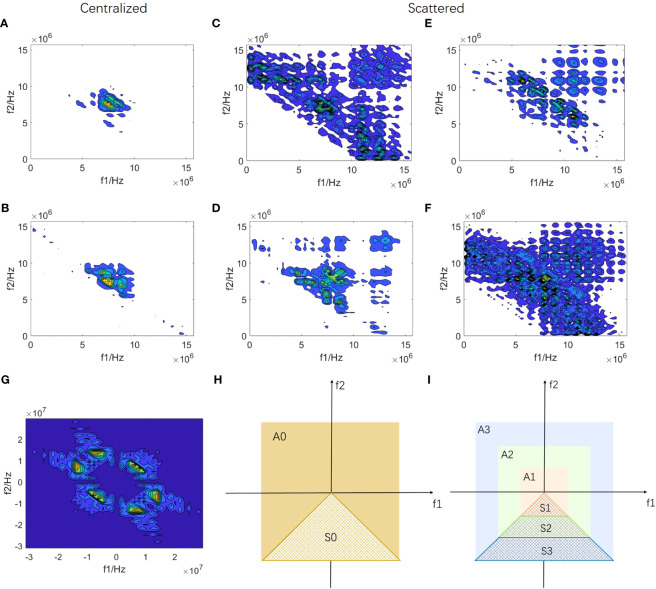
Construction of bispectrum analysis energy features **(A-F)** are local bispectrum analysis maps of the central region of six breast tumors. **(G)** is a complete bispectrum analysis map of RF signal segment *x*_bs_(*n*_bs_). A0 region of **(H)** represents the diagram of the complete bispectrum analysis, and the non-overlapping region S0 is marked. The S0 region in **(I)** is divided into three equal parts starting from the origin along the direction of the f_2_ axis, obtaining the S1, S2, and S3 regions, respectively. The bispectrum analysis energies of the non-overlapping regions of the S1, S2, S3, and S0 regions are calculated respectively to form four new bispectrum analysis energy features of RF signal segment *x*_bs_(*n*_bs_).

**Figure 1** shows the local bispectrum analysis maps of the central region of six breast tumors. They exhibit differences in distribution and have regularity. Some patients’ maps, like **Figures 1A, B** display centralized patterns, whereas others, such as **Figures 1C-F** show scattered patterns. The scattered mode indicates a wide frequency distribution of echo signals. Both scattered and centralized maps contain various rich distribution patterns. For instance, **Figures 1C, D** showcase obvious second harmonic components. In other maps, although there may not be evident second harmonics, significant distribution differences exist for other frequency components around the center frequency. These differences in frequency component distribution within local bispectrum analysis maps are closely linked to the internal microstructure of breast tumors, potentially aiding in breast cancer detection.

Based on the distribution characteristics mentioned above, we conducted frequency subdivision in the bispectrum analysis map to design four new features for each RF signal segment *x*_bs_(*n*_bs_), representing four different frequency components. **Figure 1G** illustrates the complete bispectrum analysis map with overlaps. The A0 region in **Figure 1H** represents the complete bispectrum analysis diagram, with a non-overlapping region S0 labeled. In **Figure 1I**, the diagram is divided into A1, A2, and A3 regions, with equal divisions in the horizontal direction. The overlap between S0 and A1 forms S1 (a low-frequency non-overlapping region), and the overlap between S0 and A3 forms S3 (a high-frequency non-overlapping region). S0 and A2 overlap to form S2, which encompasses a portion of both low-frequency and high-frequency components. The S0 region includes all frequency components of the S1, S2, and S3 regions. By calculating the energy of S1, S2, S3, and S0 regions, we obtain four new bispectrum analysis energy features for each RF signal segment *x*bs(*n*bs). Subsequently, we extract these four new bispectrum analysis energy features from all RF signal segments in the key frame for each patient, resulting in each patient’s four bispectrum analysis energy feature maps: BS_S1, BS_S2, BS_S3, and BS_A.

**Figure 2:**
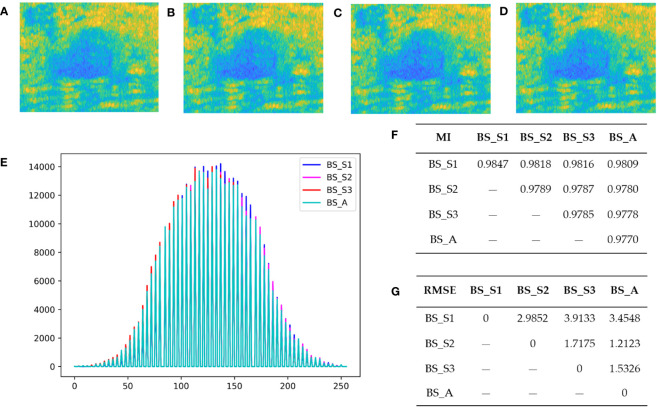
Bispectrum analysis energy feature maps of a benign patient and their differences analysis. **(A–D)** are four types of bispectrum analysis energy feature maps of a benign patient with breast tumor. **(E)** The overlapping histogram results of four bispectrum analysis energy feature maps of BS_S1, BS_S2, BS_S3, and BS_A. **(F)** The mutual information (MI) between each two of the four new bispectrum analysis energy feature maps. **(G)** The root mean square error (RMSE) between each two of the four new bispectrum analysis energy feature maps.

**Figure 3:**
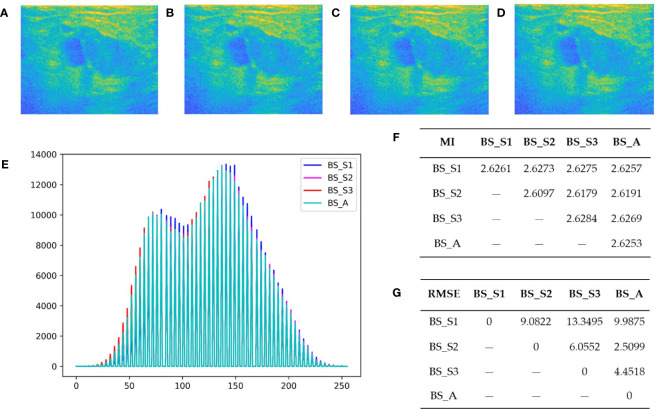
Bispectrum analysis energy feature maps of a malignant patient and analysis of their differences. **(A-D)** are four types of bispectrum analysis energy feature maps of a malignant patient with breast tumor. **(E)** The overlapping histogram results of four bispectrum analysis energy feature maps of BS_S1, BS_S2, BS_S3, and BS_A. **(F)** The mutual information (MI) between each two of the four new bispectrum analysis energy feature maps. **(G)** The root mean square error (RMSE) between each two of the four new bispectrum analysis energy feature maps.

**Figures 2A-D** display the bispectrum analysis energy feature maps BS_S1, BS_S2, BS_S3, and BS_A of a benign patient, respectively. Similarly, **Figures 3A-D** show the bispectrum analysis energy feature maps BS_S1, BS_S2, BS_S3, and BS_A of a malignant patient, respectively. Although visually similar, a closer examination of image details revealed subtle differences among the four feature maps for each patient. To quantitatively analyze these differences and correlations, gray histograms, mutual information (MI), and root mean square error (RMSE) were utilized. For a benign patient, **Figures 2E, G** depicted the statistical results of gray histograms, MI, and RMSE for the four bispectrum analysis energy feature maps. The histogram provided a visual representation of pixel value distribution, with BS_A being mostly covered by the histograms of BS_S1 (blue highlighted areas), BS_S2 (rose highlighted areas), and BS_S3 (red highlighted areas). The feature maps after frequency subdivision exhibited regular pixel distribution characteristics. The RMSE in **Figure 2G** indicated the similarity between feature maps, with higher values suggesting greater distinctiveness and necessity for classification. The average RMSE between BS_S1 and the other three feature maps was 3.451, whereas the average RMSE between BS_S3 and the other three feature maps was 2.388. Both BS_S1 and BS_S3 had higher average RMSE compared with BS_S2 (1.972) and BS_A (2.067), highlighting their representativeness and importance. MI in **Figure 2F** measured the strength of the relationship between random variables. The average MI between BS_S1 and the other three feature maps was the highest at 0.981, followed by BS_S3 at 0.979. This indicated that BS_S1 and BS_S3 contained the majority of information from the other feature maps.

In summary, the quantitative analysis results demonstrated that BS_S1 and BS_S3 provided greater information content and differences compared with BS_A and BS_S2. Similar observations were made for malignant patients, where BS_S1 represented the low-frequency energy and BS_S3 represented the high-frequency energy. Frequency subdivision contributed to enhanced information richness and value of bispectrum analysis energy feature maps, enabling a more comprehensive analysis of microscopic and macroscopic tissue information.

### Techniques of RF-based deep learning classification network

#### Overall design of network framework

We proposed the deep learning method based on bispectrum analysis energy feature maps of ultrasound RF signals to detect breast cancer. The overall design of the final deep learning network framework is depicted in **Figure 3**. In our proposed deep learning method, a weight sharing network framework was designed for input of multiple feature maps. To enhance the importance of advantageous feature maps in classification tasks, a feature map attention module was implemented. Additionally, we designed a similarity constraint factor module to calculate cosine distances and learn the similarities and differences between different feature maps.

#### Weight sharing network framework

Weight sharing is crucial for extracting diverse sample features while minimizing network complexity. It greatly reduces the number of network parameters and the computational complexity during learning. Weight sharing can be manifested in various ways, such as sharing convolution kernel weights or weights of the entire network module [18,19]. A well-designed weight sharing structure enhances network depth, efficiency, and a lightweight architecture.

**Figure 4:**
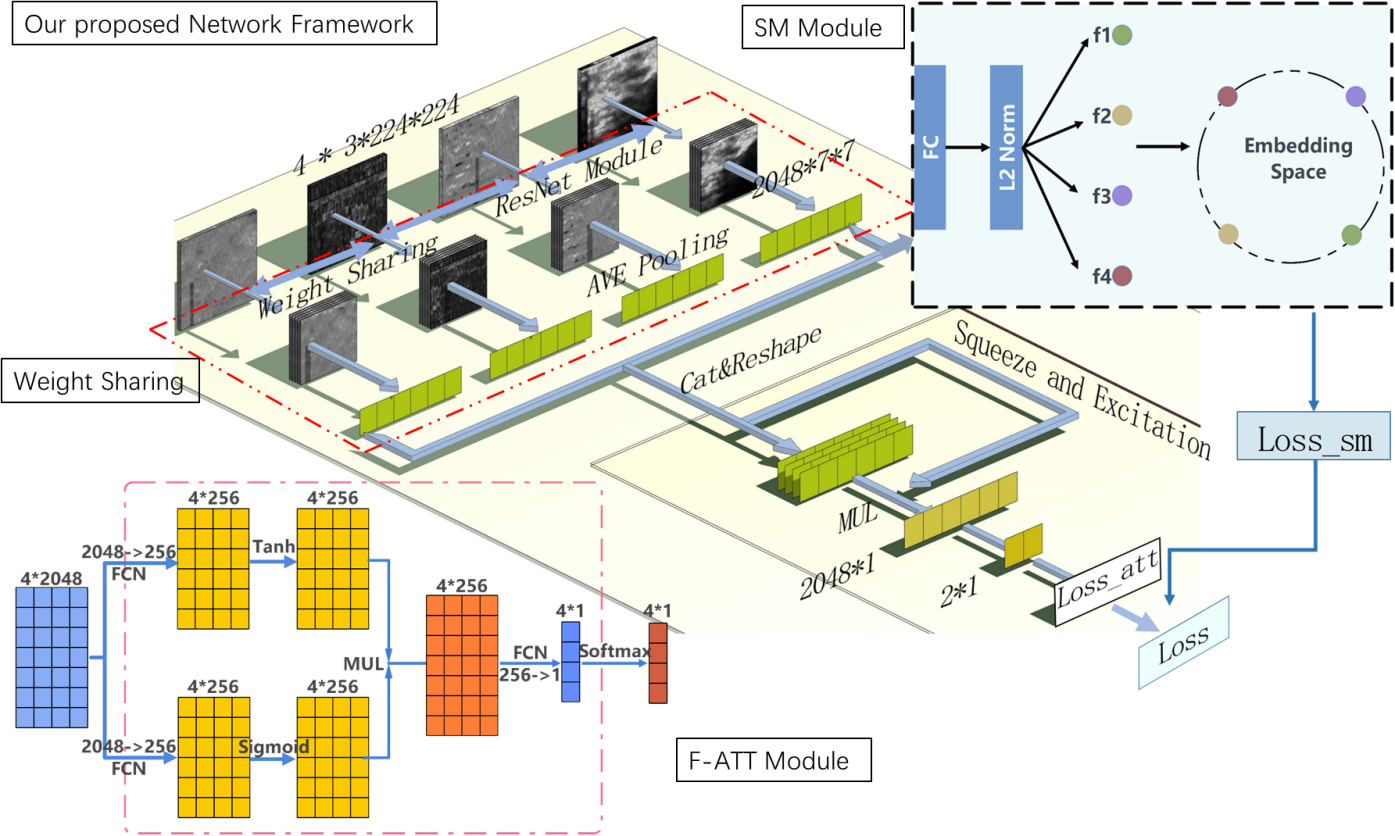
Overall design of our proposed method of deep learning model framework. Diagram of our proposed method of the deep learning model based on RF signals bispectrum analysis energy feature maps. Its backbone network is ResNet-50 and includes weight sharing module, feature map attention (F-ATT) module, and similarity constraint factor (SM) module.

In this study, we established a four-channel weight sharing deep learning network that shares weights across the entire backbone network. **Figure 4** illustrates the structural diagram of this weight sharing network framework. The backbone network was selected from popular modules such as Swin Transformer, VGG-19, Inception-v3, ResNet50, and ResNet-101. The network with the highest performance on the experimental dataset was chosen as the backbone network of the weight sharing module. The 4-channel network shares weights, enabling it to learn common features from all input images while preserving individual characteristics.

#### Feature maps attention module

To prevent excessive features from hindering network optimization efficiency in small volume medical data, we introduced an attention mechanism. This mechanism adaptively reconstructs the importance of each feature map, prioritizing the ones beneficial to the target task. The attention mechanism adjusts the weighting model based on specific learning tasks, strengthening relevant content and disregarding irrelevant features. Since the importance of the four feature maps in classifying benign and malignant breast tumors varies, it is crucial to assess their contributions for convenient and rapid tumor classification in clinical applications. Hence, we designed a feature map attention module based on attention mechanism (F-ATT), which was integrated into the weight sharing network framework.

The F-ATT module employed a weight sharing network feature processing method based on self-attention mechanism. As shown in equation 3, during the training process, the network adaptively learned the weight parameters corresponding to the feature vectors of the four types of feature maps, and then these feature vectors were weighted and summed. The sum of the weights of the feature vectors was 1, ensuring that the dimension of the feature values did not change. Moreover, the size of these weights represented the role of the feature vector in the final classification. The higher the weight, the greater the impact on the classification of benign and malignant breast tumors.


(3) $${\text{z}}_{\text{dl}}=\sum_{k=1}^{K}a_{k}h_{k}$$

Among them, *h_k_* represents the vector mapping of the kth instance in a package, z_dl_ represents the weighted average of its various instances, and *a_k_* represents the weight of each instance learned by network adaptation; the calculation formula is as follows:


(4) $$\alpha_{k}=\frac{\exp\left\{w^{T}\left(tanh\left(Vh_{k}^{T}\right)\;\cdot \;sigm\left(Uh_{k}^{T}\right)\right)\right\}}{\sum_{j=1}^{K}exp\left\{w^{T}\left(tanh\left(Vh_{j}^{T}\right)\;\cdot \;sigm\left(Uh_{j}^{T}\right)\right)\right\}}$$

Among them, $V、U\in {\mathbb{R}}^{LXM}$ is the point multiplication operation, *w^T^* is the weight vector, and *T* is the transpose operation.

The activation function is the key to realizing the network non-linear classification task. The diagram of the attention mechanism structure established in this study is shown in **Figure 4**; an activation unit based on the gate mechanism was used.

Combining the tanh activation function with the sigmoid activation function can weaken the approximately linear influence in the tanh activation function. Perform tanh nonlinear activation and sigmoid nonlinear activation on the feature vectors respectively, then multiply the output feature values of the two by their corresponding elements, and then connect them to a fully connected layer to output the obtained weights. Finally, the softmax function was used to convert the weights into weight parameters where each term was positive and the sum was 1. Through the above feature map selection mechanism, the weight of feature maps that were not important to the classification task was reduced, and redundant feature maps were automatically eliminated to filter out feature maps that contributed to the classification.

The Loss function of the weight sharing backbone network based on the feature map attention module was Loss_att. The calculation formula for function is as follows:


(5) $$\text{Loss}\_\text{att}=-\frac{1}{N_{t}}\sum_{i_{t}}\left[y_{i_{t}}\cdot \log\left(p_{i_{t}}\right)+\left(1-y_{i_{t}}\right)\cdot \log\left(1-p_{i_{t}}\right)\right]$$

Among them, $y_{i_{t}}$ represents the sample Label of *i_t_*, $p_{i_{t}}$ represents the probability of a negative prediction, $1-p_{i_{t}}$ represents the probability of a positive prediction, and *N_t_* represents the number of samples.

#### Similarity constraint factor

Cosine similarity has been effective in addressing high-dimensional Euclidean distance challenges and has demonstrated positive outcomes in various practical applications. For instance, in self-supervised contrastive learning, cosine distance calculation plays a vital role in achieving unlabeled network learning tasks by comparing positive and negative sample pairs. In this study, we employed cosine similarity to construct a similarity constraint factor module (SM) for bispectrum analysis feature maps. By calculating the vector angle between feature maps, the module evaluates their similarity and dissimilarity.

The formula for measuring the distance between positive and negative sample pairs using cosine similarity is as follows:


(6) $$cos\theta \;=\;\frac{f_{i}\cdot f_{j}}{\left||f_{i}||\;\;||f_{j}|\right|}=f_{i}\cdot f_{j}$$

Among them, ***f_i_*** and ***f_j_*** represent any two of the feature value vectors output by the backbone network and have undergone *L*2 normalization processing.

**Figure 5:**
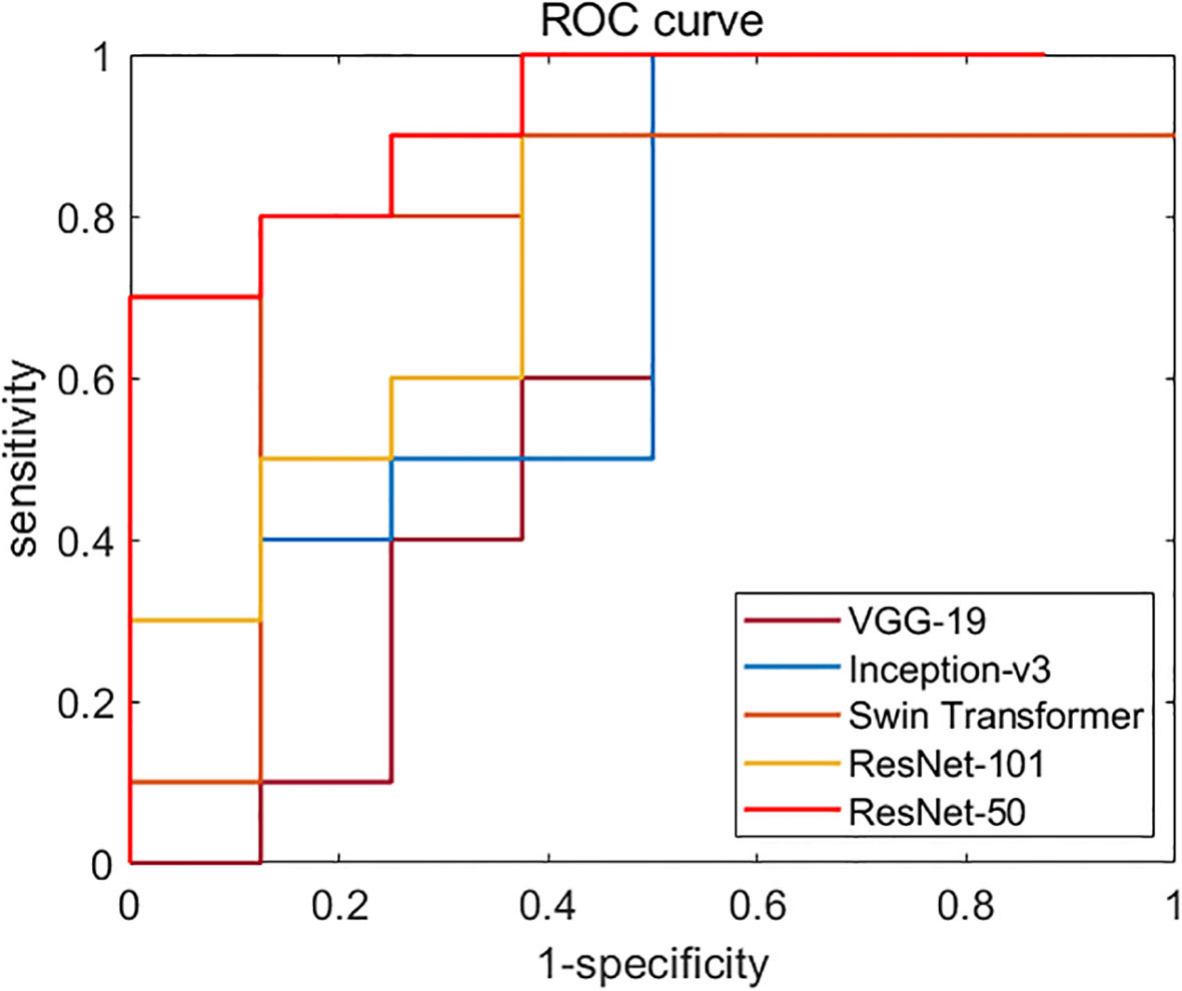
Diagnostic performances of different backbone network models. ROC curves of VGG-19, Inception-v3, ResNet-50, ResNet-101, and Swin Transformer network. The AUC of ResNet-50 (test set 1, 0.819; test set 2, 0.731) was the highest one among all models.


The input of the SM module was the output of the weight sharing network module based on four bispectrum analysis energy feature maps, which was the same as the input of the F-ATT module. $x_{i_{m}}$ and $x_{j_{m}}$ represent the *i_m_* and *j_m_* feature maps. We hoped that the probability of $x_{i_{m}}$ being recognized as *i_m_* class was as high as possible, whereas the probability of $x_{j_{m}}$ being recognized as *i_m_* was as low as possible, achieving the goal of positive concentration and negative separation. Because the deep learning optimizer was designed with minimum optimization, the sum of negative logarithms was used to design the Loss function *Loss_sm* of the SM module. *Loss_sm* is shown in **Figure 5**, and its calculation formula is as follows:

(7) $$\text{Los}s\_sm=-\sum_{i_{m}}\log P\left(i_{m}x_{i_{m}}\right)-\sum_{i_{m}}\sum_{j_{m}\neq i_{m}}\log\left(1-P\left(i_{m}x_{j_{m}}\right)\right)$$

Among them, $P\left(i_{m}x_{i_{m}}\right)$ represents the probability that $x_{i_{m}}$ is recognized as class *i_m_*; $P\left(i_{m}x_{j_{m}}\right)$ represents the probability of $x_{j_{m}}$ being recognized as a class *i_m_*. The specific derivation and calculation process of both are detailed in reference (21).

### Parameter setting and training process

In this study, the sample’s four maps underwent scaling and center cropping according to the designed data enhancement rules. Each input map was initially scaled to 3 × 256 × 256 and then cropped to 3 × 224 × 224. The training set, validation set, and two test sets’ data were shuffled randomly and fed into the network for training. Typically, a batch size of 8 was used when reading images. The Adam optimizer and the cosine annealing attenuation strategy were employed, starting with an initial learning rate of 0.002. The learning rate was adjusted using the CosineAnnealingLR strategy, with a final learning rate of 1e-4. Training was concluded, and the results were saved when the validation set’s loss ceased to decrease and remained stable.

The model was implemented using a Python-based framework, utilizing an Intel Xeon Silver 4216 CPU running at 2.10 GHz and an NVIDIA A10 GPU based on the Nvidia Ampere framework.

### Statistical analysis

AUC, accuracy, sensitivity, specificity, positive predictive value (PPV), and negative predictive value (NPV) were used to evaluate the classification performance of the model. The calculation formulas for accuracy, sensitivity, specificity, PPV, and NPV are shown in equations (8), (9), (20), (11) and (12):


(8) $$\text{accuracy}=\frac{\text{TP+TN}}{\text{TP}+\text{TN}+\text{FP}+\text{FN}}$$

(9) $$\text{sensitivity}=\frac{\text{TP}}{\text{TP}+\text{FN}}$$

(10) $$\text{specificity}=\frac{\text{TN}}{\text{TN}+\text{FP}}$$

(11) $$\text{PPV}=\frac{\text{TP}}{\text{TP}+\text{FP}}$$

(12) $$\text{NPV}=\frac{\text{TN}}{\text{TN}+\text{FN}}$$

where TP, FP, TN, and FN represent the number of true positive patients, false positive patients, true negative patients, and false negative patients in the classification results, respectively.

In addition, this study also established a deep learning classification model based on ultrasound grayscale image as a comparative experiment.

## Results

### Result of backbone network

We first compared the classification performances of the five backbone models based on four bispectrum analysis energy feature maps: VGG-19, Inception-v3, ResNet-50, ResNet-101, and Swin Transformer. We then selected the appropriate backbone network for the weight sharing network framework as the feature extractor of RF signals. **Figure 5** displays the ROC curves of these backbone networks on independent test set 1. Among the five networks, ResNet-50 exhibited the highest performance with an AUC of 0.819, making it the chosen backbone network for the weight sharing framework in our deep learning model based on ultrasound multiple feature maps of RF signals.

With ResNet-50 selected as the backbone network, traditional data augmentation methods were employed to preprocess the four feature maps and enhance the generalization performance of our deep learning models. The size of each ultrasound feature map was adjusted to 3 × 224 × 224. Subsequently, the weight sharing network module based on pretrained ResNet-50 was trained for each feature map type, extracting 2,048 × 7 × 7-dimensional feature maps. Through averaging and pooling, we obtained 2,048 × 1-dimensional features from the backbone network.

These 2,048 × 1-dimensional weight features from the four ultrasound feature maps facilitated weight sharing within the ResNet-50 network. The front-end processing framework of the weight sharing module, in conjunction with the F-ATT and SM modules, constituted our proposed deep learning model based on the four feature maps of RF signals.

### Classification result and comparative experiments

**Table 1** displays the classification result of our proposed deep learning model based on four bispectrum analysis energy feature maps. The classification results of the comparison model based on ultrasound grayscale image and the comparison models using single bispectrum analysis energy feature map are also shown in **Table 1**. Both the comparison model based on grayscale image and comparison model based on single bispectrum analysis energy feature map comparison models were all single feature map input, so their network structures were the same. We modified the structure of our proposed network model based on four bispectrum analysis energy feature maps to adapt to single input. The model still used the ResNet-50 network to extract features from the input image, resulting in a 2,048 × 1-dimensional feature vector. The F-ATT module no longer generated feature map coefficients, but directly processed the 2,048 × 1-dimensional feature vector above. The final comparison model no longer contained the SM module due to single image input.

**Table 1:** Classification performances of our proposed model and various comparative models.

Model	Data Type	AUC	Accuracy (%)	Sensitivity (%)	Specificity (%)	PPV (%)	NPV (%)
Our proposed model	Validation Set	0.913	83.33	80.00	87.50	88.89	77.78
	Test Set 1	0.900	77.78	90.00	62.50	75.00	83.33
	Test Set 2	0.885	80.00	95.00	65.00	73.08	92.86
BS_S1 feature map	Validation Set	0.800	77.78	80.00	75.00	80.00	75.00
	Test Set 1	0.813	77.78	70.00	87.50	87.50	70.00
	Test Set 2	0.796	76.25	81.25	68.75	79.59	70.97
BS_S2 feature map	Validation Set	0.825	72.22	90.00	50.00	69.23	80.00
	Test Set 1	0.738	66.67	60.00	75.00	75.00	60.00
	Test Set 2	0.732	66.25	62.50	71.87	76.92	56.10
BS_S3 feature map	Validation Set	0.875	83.33	90.00	75.00	81.82	85.71
	Test Set 1	0.775	77.78	80.00	75.00	80.00	75.00
	Test Set 2	0.855	75.00	66.67	87.50	88.89	63.64
BS_A feature map	Validation Set	0.863	77.78	90.00	87.50	87.50	70.00
	Test Set 1	0.725	72.22	70.00	75.00	77.78	66.67
	Test Set 2	0.786	73.75	85.42	56.25	74.55	72.00
ultrasound grayscale image	Validation Set	0.750	72.22	60.00	87.50	85.71	63.64
	Test Set 1	0.713	61.11	40.00	87.50	80.00	53.85
	Test Set 2	0.694	60.00	42.50	77.50	65.38	57.41

AUC, area under the receiver operating characteristic curve; PPV, positive predictive value; NPV, negative predictive value.

Our proposed deep learning model, incorporating four new bispectrum analysis energy feature maps based on RF signals, consistently maintained an AUC of 0.900 or higher on both the validation set and test set 1. The sensitivity on test set 1 reached 90.00%, indicating the model’s ability to effectively detect malignant breast tumors and reduce the risk of missed diagnoses. To assess generalization performance, we introduced an independent test set 2. Even on new data collected on different dates, our proposed model exhibited strong classification performance, achieving an AUC of 0.885 for classifying benign and malignant breast tumors, demonstrating excellent stability.

Among the four comparison models utilizing single bispectrum analysis energy feature maps, the models employing the low-frequency energy feature map of BS_S1 and the high-frequency energy feature map of BS_S3 demonstrated better performance. The average classification AUC of the model utilizing the BS_S3 feature map was 0.815 across test set 1 and test set 2. Nonetheless, the model utilizing all four bispectrum analysis energy feature maps outperformed the models utilizing a single feature map.

Additionally, the AUC of the comparison model based on ultrasound grayscale images was 0.713 and 0.694 on the two independent test sets, respectively. Compared with this, our proposed model based on four bispectrum analysis energy feature maps exhibited an increase in AUC of 0.225 and 0.191 on the respective independent test sets. Our proposed deep learning model based on ultrasound RF signals demonstrated a higher level of differentiation ability between benign and malignant breast tumors than the deep learning model based on ultrasound grayscale image.

### Ablation experiment results

Our proposed model was tailored to the characteristic of multiple inputs of bispectrum analysis energy feature maps. In addition to the selection of backbone network of the weight sharing module, the model also included the F-ATT and SM modules. To verify the rationality and effectiveness of our model design, we conducted ablation experiment. The results are shown in **Table 2**.

**Table 2:** Classification results of ablation experiment.

ResNet-50	Weight Sharing	F-ATT	SM	Data Type	AUC	Accuracy (%)	Sensitivity (%)	Specificity (%)	PPV (%)	NPV (%)
√				Validation Set	0.775	72.22	70.00	75.00	77.78	66.67
				Test Set 1	0.819	83.33	100.00	62.50	76.92	100.00
				Test Set 2	0.703	66.25	70.00	62.50	65.12	67.57
√	√			Validation Set	0.788	72.22	70.00	75.00	77.78	66.67
				Test Set 1	0.813	72.22	90.00	50.00	69.23	80.00
				Test Set 2	0.731	70.00	72.50	67.50	69.05	71.05
√	√	√		Validation Set	0.825	77.78	70.00	87.50	87.50	70.00
				Test Set 1	0.863	77.78	80.00	75.00	80.00	75.00
				Test Set 2	0.868	78.75	70.00	87.50	84.85	74.47
√	√	√	√	Validation Set	0.913	83.33	80.00	87.50	88.89	77.78
				Test Set 1	0.900	77.78	90.00	62.50	75.00	83.33
				Test Set 2	0.885	80.00	95.00	65.00	73.08	92.86

F-ATT, feature map attention Module; SM, similarity constraint factor module.

When our proposed method did not use weight sharing and F-ATT and SM modules, it was also the ResNet-50 model as shown in **Figure 5**. The classification performance of the model was the lowest among the four models in the ablation experiment. When our proposed method removed F-ATT and SM modules and only retained the weight sharing framework, although there was no significant improvement, weight sharing reduced the number of parameters. Compared with the model without weight sharing and establishing four independent ResNet-50 networks to extract features from four input feature maps, our proposed method had weight sharing, with only 25.83% of the former’s parameter count. When our proposed method used the weight sharing framework and F-ATT module, and only removed SM modules, the AUC results of test set 1 and test set 2 increased by 0.044 and 0.165, respectively. When both weight sharing and F-ATT and SM modules were introduced, our proposed method in this study was formed. Compared with the initial ResNet-50 model, our final model increased the AUC of the test set 1 and test set 2 by 9.9% and 25.89%, respectively.

### Analysis of the importance of feature maps

**Figure 6:**
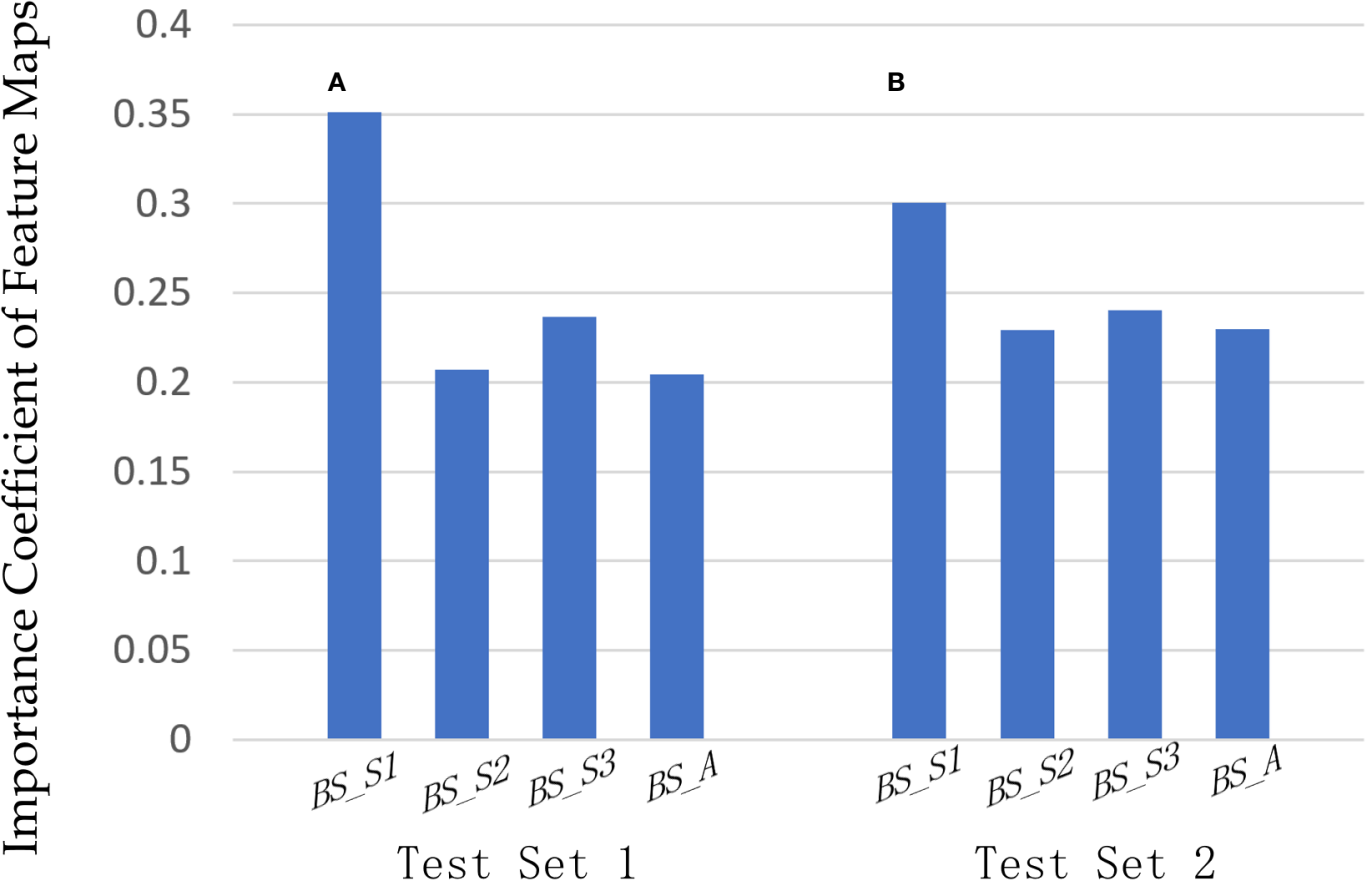
Importance analysis of four bispectrum analysis energy feature maps. The importance coefficients of the four bispectrum analysis energy feature maps on **(A)** independent test set 1 and **(B)** independent test set 2, outputting by the F-ATT module.

In our proposed method based on bispectrum analysis energy feature maps of RF signals, the F-ATT module dynamically learned the influence of multiple input feature maps on classification and adjusted the weights of feature maps based on loss. **Figure 6** illustrates the importance coefficients outputted by the F-ATT module for the four bispectrum analysis energy feature maps across two independent test sets. **Figure 6A** represents the importance of the four bispectrum analysis feature maps on independent test set 1, whereas **Figure 6B** represents the importance of the four bispectrum analysis feature maps on independent test set 2.

On independent test set 1, the importance coefficients for the four bispectrum analysis energy feature maps BS_S1, BS_S2, BS_S3, and BS_A were 0.3513, 0.2073, 0.2368, and 0.2045, respectively. The BS_S1 feature map primarily captured the bispectrum analysis energy of low-frequency regions and had the highest importance coefficient for classifying benign and malignant breast tumors. The second most important feature map was BS_S3, which focused on the bispectrum analysis energy of high-frequency regions. The importance of feature map BS_S1 in the low-frequency region and feature map BS_S3 in the high-frequency region for classification was higher than that of feature map BS_A in full-frequency regions. The BS_S2 feature map encompassed parts of both high-frequency and low-frequency regions, and its importance for classification was not as significant as that of BS_S1 and BS_S3. The same trend was observed on independent test set 2 in **Figure 6B**, where BS_S1 and BS_S3 remained the two most important feature maps for classifying benign and malignant breast tumors.

## Discussion

RF signals, obtained directly from ultrasound diagnostic equipment without undergoing processing such as envelope detection and contrast adjustment, offer richer tissue information compared with traditional ultrasound images. Exploring and developing the utilization efficiency of RF signals is crucial. However, due to the presence of noise and interference components, directly applying traditional machine learning or deep learning techniques to RF signals is challenging for achieving efficient applications. To address this, we devised multiple feature maps and then modeled them based on deep learning to achieve efficient feature extraction of RF signals. Our innovative approach involved designing bispectrum analysis energy feature maps specifically for ultrasound RF signals. We then proposed a deep learning method to further process these feature maps, enabling effective classification of benign and malignant breast tumors.

Ultrasound feature maps provided a more comprehensively display of the entire tumor’s feature information. Traditional methods often intercepted signals from regions of interest of tumors for feature extraction and post-processing. It ignored the heterogeneity of tumors. The tumor microenvironment usually has significant differences. Especially malignant tumors have irregular growth patterns and complex internal structures. The local tumor region is difficult to represent the overall pathological condition and the types of tumors. By combining feature maps with deep learning, multiscale, standardized, and comprehensive tumor feature extraction was achieved, mitigating the impact of tumor heterogeneity on classification outcomes. Shao et al. (20) extracted a large number of features from RF time series, including bispectrum analysis features, and used Random Forest and Support Vector Machine classifiers to identify breast cancer. The use of standard deviation features of bispectrum analysis can achieve the AUC of 86%. In our study, we further explored the bispectrum analysis features by proposed bispectrum analysis energy feature maps and designed a deep learning network model to detect breast cancer. The average AUC of our study on two independent test sets reached 0.893, which is superior to the above machine learning processing algorithm based on bispectrum analysis features.

The incorporation of bispectrum analysis energy feature maps in our study introduced high-order moments and refines feature maps from different-frequency components. These feature maps included four frequency components, making the extracted feature levels more abundant. Notably, the four feature maps exhibit varying importance levels for classification and demonstrate regularity. The statistical results of overlapping grayscale histograms, MI, and RMSE showed that the bispectrum analysis energy feature map of BS_S1 in the low-frequency region and BS_S3 in the high-frequency region had greater information content and differences for classification than BS_S2 and BS_A. Analyzing the importance coefficients of the feature map output by the F-ATT module in our proposed model, it is evident that both on independent test set 1 and independent test set 2, feature map BS_S1 in the low-frequency region and feature map BS_S3 in the high-frequency region also hold higher importance for classification compared with feature map BS_A in the full-frequency region. Moreover, this observation is also supported by the results of the comparison models based on a single bispectrum analysis energy feature map. In **Table 1**, the average AUC values of the comparison model using the single feature map of BS_S1 and the comparison model using the single feature map of BS_S3 on two independent test sets were 0.805 and 0.815, respectively. They were also higher than that of the other two comparison models using single feature maps of BS_S2 and BS_A, respectively. Importantly, although there were differences in the importance of four feature maps for classification, utilizing all four feature maps yields superior classification performance compared with using any single feature map. In addition, the AUC values of our proposed deep learning model based on four bispectrum analysis energy feature maps on two independent test sets were higher than that of the deep learning model based on grayscale images. Overall, the bispectrum analysis energy feature maps with frequency subdivision provide ample and high-quality frequency information that contributes significantly to breast cancer classification.

In this study, we proposed novel ultrasound images and processed them by the deep learning method for breast cancer detection. There are also many studies that investigated various modes of ultrasound images by deep learning for classification tasks. Qian et al. (22) developed an explainable deep learning system trained on 10,815 multimodal breast ultrasound images, achieving an AUC of 0.955 for predicting BI-RADS scores for breast cancer. Byra et al. (9) used VGG-19, Inception-v3, and ResNet V2 CNN models to process ultrasound grayscale images reconstructed by different algorithms and classify benign and malignant breast tumors, reaching a maximum AUC of 0.857. Zeimarani et al. (23) proposed a novel breast ultrasound grayscale image classification method based on deep convolutional neural networks. After applying image enhancement and regularization, the accuracy and AUC were improved to 92.01% and 0.972, respectively. Koh et al. (24) designed CNN to process ultrasound grayscale image for differentiating thyroid nodules. Their results demonstrated the diagnostic performances of CNN-based method comparable with expert radiologists for differentiating thyroid nodules on grayscale image. Kim et al. (18) employed CNN to process multiple parameter images generated from RF signals for benign and malignant breast tumor analysis, including grayscale, entropy, attenuation, and phase images. The highest accuracy and sensitivity were 83.00% and 92.24%, respectively. A specially designed deep learning architecture can fully explore the feature information that is helpful for classification in various modes of ultrasound images. The deep neural network established based on multiple ultrasound feature maps has better adaptability and development value.

With the design of feature maps, the selection of backbone network, SM module, and the addition of feature map importance screening and sorting module F-ATT, our proposed deep learning method ultimately achieved effective classification for benign and malignant breast tumors. Ablation experiments further investigated the performance and contribution of different modules, affirming the rationality of the deep learning model architecture when processing RF signals. Utilizing deep learning for processing various feature maps of ultrasound RF signals proved to be an efficient and reliable method, expected to become a conventional signal processing approach. In this study, the various modules designed for multifeature maps of RF signals had varying degrees of contribution to the classification target. The results of the validation set indicated that the SM module contributed the most to the improvement of classification results. The average sensitivity of two independent test sets reached 92.50%, reducing the occurrence of missed diagnosis. Weight sharing demonstrated significant contributions to model lightweighting. For multi-input feature maps, weight sharing reduced parameters and computational costs without compromising network performance. Previous studies, such as Zheng et al. (25) and Aich et al. (26), also highlighted the benefits of weight sharing, achieving similar performance while reducing parameters. In addition, the F-ATT module in our proposed method facilitated clear and intuitive learning of the importance levels of each feature map for the designated classification task, enhancing visualization. Comparing the importance of four bispectrum analysis feature maps and ultrasound grayscale images, we observed that the low-frequency component had the greatest impact on classification, followed by the high-frequency component, both being more important than the full-frequency component. This study showcased the advantages of frequency refinement processing in bispectrum analysis. Furthermore, the inclusion of the F-ATT module in the weight sharing ResNet-50 network improved evaluation indicators, including AUC, accuracy, specificity, and PPV, on the validation and two test sets. The self-attention mechanism not only improved classification results but also adjusted feature map importance, reducing redundancy and achieving a lightweight and efficient model.

To ensure reliable classification of benign and malignant breast tumors, we evaluated the network performance on two independent test sets, although the data were from a single center. To enhance the generalization capabilities, our future steps involve collecting data from multiple centers. Furthermore, we aim to develop additional feature maps based on RF signals, conduct extensive performance analysis, and further enhance the classification of breast tumor benign and malignant cases.

In summary, our study introduced novel bispectrum analysis energy feature maps with frequency subdivision for ultrasound RF signals. Through our designed deep learning framework, we achieved effective differentiation of benign and malignant breast tumors, enhancing the utilization efficiency of ultrasound RF signals.

## Data availability statement

The raw data supporting the conclusions of this article will be made available by the authors, without undue reservation.

## Ethics statement

The studies involving humans were approved by Ruijin Hospital Ethics Committee Shanghai JiaoTong University School of Medicine. The studies were conducted in accordance with the local legislation and institutional requirements. Written informed consent for participation in this study was provided by the participants’ legal guardians/next of kin. Written informed consent was obtained from the individual(s) for the publication of any potentially identifiable images or data included in this article.

## Author contributions

QW: Data curation, Investigation, Methodology, Software, Writing – original draft. XJ: Conceptualization, Data curation, Methodology, Writing – review & editing. TL: Data curation, Supervision, Validation, Writing – review & editing. JY: Conceptualization, Methodology, Supervision, Writing – review & editing. SX: Conceptualization, Methodology, Supervision, Validation, Writing – review & editing.
